# Crystallographic Analysis of Polypyrimidine Tract-Binding Protein-Raver1 Interactions Involved in Regulation of Alternative Splicing

**DOI:** 10.1016/j.str.2011.09.020

**Published:** 2011-12-07

**Authors:** Amar Joshi, Miguel B. Coelho, Olga Kotik-Kogan, Peter J. Simpson, Stephen J. Matthews, Christopher W.J. Smith, Stephen Curry

**Affiliations:** 1 Division of Cell and Molecular Biology, Imperial College, Exhibition Road, London SW7 2AZ, UK; 2 Division of Molecular Biosciences, Imperial College, Exhibition Road, London SW7 2AZ, UK; 3 Department of Biochemistry, University of Cambridge, Tennis Court Road, CB2 1QW, UK

## Abstract

The polypyrimidine tract-binding protein (PTB) is an important regulator of alternative splicing. PTB-regulated splicing of α-tropomyosin is enhanced by Raver1, a protein with four PTB-Raver1 interacting motifs (PRIs) that bind to the helical face of the second RNA recognition motif (RRM2) in PTB. We present the crystal structures of RRM2 in complex with PRI3 and PRI4 from Raver1, which—along with structure-based mutagenesis—reveal the molecular basis of their differential binding. High-affinity binding by Raver1 PRI3 involves shape-matched apolar contacts complemented by specific hydrogen bonds, a new variant of an established mode of peptide-RRM interaction. Our results refine the sequence of the PRI motif and place important structural constraints on functional models of PTB-Raver1 interactions. Our analysis indicates that the observed Raver1-PTB interaction is a general mode of binding that applies to Raver1 complexes with PTB paralogues such as nPTB and to complexes of Raver2 with PTB.

## Highlights

► First crystal structures of PTB reveals how it binds Raver1 via PRI motifs ► Observed mode of binding is general for PRIs and PTB paralogues ► Results place constraints on models of splicing repression by PTB-Raver1 complexes

## Introduction

Alternative splicing in metazoans produces multiple messenger RNA (mRNA) transcripts from a single gene and is a powerful mechanism for amplifying proteome complexity. Over 95% of human multiexon genes have multiple splice isoforms (Nilsen and Graveley, 2010). The process of pre-mRNA splicing involves the controlled inclusion or exclusion of specific exons and is regulated by *cis*-acting enhancer or silencer sequences found either within the exon or within the flanking introns (Matlin et al., 2005). The temporal and spatial control of splicing is also determined by various positive and negative protein cofactors, which are activated by developmental or differentiation-specific cues.

One of the most intensively studied regulators of alternative splicing is the polypyrimidine tract-binding protein (PTB). PTB is a versatile protein. In addition to its nuclear splicing activity, PTB is also found in the cytoplasm, where it has roles in the stabilization and localization of mRNA (Ghetti et al., 1992; Cote et al., 1999; Tillmar et al., 2002). It is also recruited to stimulate translation initiation driven by internal ribosome entry sites from cellular and viral mRNAs (Sawicka et al., 2008).

PTB is expressed in a variety of tissues (Patton et al., 1991) and represses many muscle and neuron-specific exons, tissues in which PTB levels are low (Xue et al., 2009; Llorian et al., 2010). It has a number of tissue-specific paralogues in neurons (nPTB) (Markovtsov et al., 2000; Polydorides et al., 2000), hematopoietic cells (ROD1) (Yamamoto et al., 1999) and smooth muscle cells (smPTB) (Gooding et al., 2003) which, with at least 50% amino acid sequence identity, are closely related to the prototypical protein. These paralogues appear to supplant or modulate the program of splicing activity of PTB in the tissues where they occur (Boutz et al., 2007; Makeyev et al., 2007).

Though best known as a negative regulator of splicing that acts to exclude specific exons, PTB has recently been found to determine exon inclusion in some cases (Xue et al., 2009; Llorian et al., 2010). In common with a number of other splicing regulators, its activity appears to be determined by the location at which it binds to the RNA relative to the regulated exon (Witten and Ule, 2011).

Several different mechanisms have been proposed for the repressive splicing activity of PTB, including direct binding to pre-mRNA in order to block binding of U2AF65—a component of the spliceosome—to the polypyrimidine tract (Lin and Patton, 1995; Singh et al., 1995) or oligomeric assembly across exons to mask splice sites (Wagner and Garcia-Blanco, 2001). In other cases, PTB does not directly block splicing factor binding, but rather the formation of splicing complexes across exons or introns; these indirect mechanisms appear to result from the ability of PTB to remodel pre-mRNA by bringing distal regions of RNA into close proximity and so inducing looping (Chou et al., 2000; Izquierdo et al., 2005; Cherny et al., 2010) or, as has been shown more recently, by bridging nonproductive association of pre-mRNA with stem-loop IV of U1 small nuclear RNA (snRNA) (Sharma et al., 2008; Sharma et al., 2011). These mechanisms may all operate, depending on the particular pre-mRNA being spliced, though part of the observed variety is likely a reflection of the technical difficulty of studying splicing at the molecular level.

**Figure 1:**
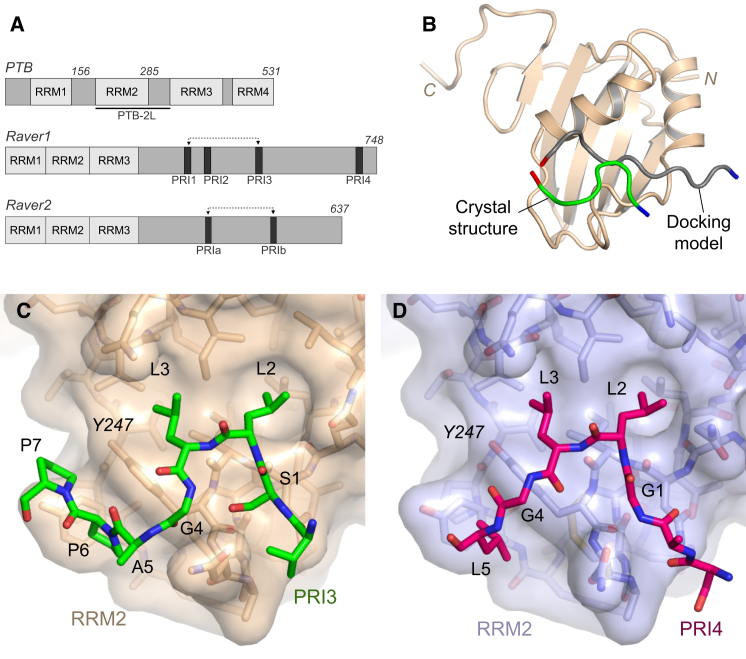
Structures of Raver1 PRIs Bound to PTB RRM2 (A) Schematic diagrams of PTB, Raver1, and Raver2 showing locations of RRMs and PRIs. Residue numbers are indicated above each protein. (B) Comparison of the crystal structure of Raver1 PRI3 (green) bound to RRM2 (tan) with the NMR-restrained docking model of the PRI3 peptide (gray). The structures are shown schematically as cartoon representations; the N and C termini of the peptides are colored blue and red, respectively. (C and D) Close-up views of the interactions of (C) Raver1 PRI3 and (D) Raver1 PRI4 with PTB RRM2. The Van der Waals surface of the RRM is depicted as a semitransparent skin. All structural figures were created using PyMOL (Delano, 2002). See also Figure S1.

Nevertheless, it is clear that PTB binds to both RNA and proteins during splicing. The protein binds pyrimidine-rich motifs within RNA (e.g., UCUU, CUCUCU), which are found in regulatory elements (Pérez et al., 1997; Ray et al., 2009) that may be structured (Clerte and Hall, 2009; Kafasla et al., 2009). PTB binds RNA via β sheet surfaces on the four RNA recognition motif domains (RRMs; Figure 1A) (Conte et al., 2000; Simpson et al., 2004; Oberstrass et al., 2005) that are arrayed in an elongated conformation (Petoukhov et al., 2006). This arrangement allows multipoint contacts with RNA targets that can remodel or stabilize RNA structures containing pyrimidine-rich motifs and offers a plausible basis for models of PTB-mediated repression that invoke looping of pre-mRNA (Oberstrass et al., 2005; Cherny et al., 2010; Lamichhane et al., 2010) or contacts between pre-mRNA and U1 snRNA (Sharma et al., 2011).

Splicing regulation by PTB or its paralogues also appears to involve interactions with other regulatory proteins, including Nova-1 and Nova-2 (Polydorides et al., 2000), Raver1 (Gromak et al., 2003), and MRG15 (Luco et al., 2010). The best characterized of these is the PTB-Raver1 interaction, which modulates splicing of α-tropomyosin (*Tpm1*). PTB acts to exclude the mutually exclusive exon 3 of *Tpm1* in smooth muscle cells but not in other cells where PTB is expressed (Gooding et al., 1998). Overexpression of PTB has little effect on this splicing event, suggesting that it is not limiting. However, overexpression of Raver1 (Hüttelmaier et al., 2001) causes a large increase in exon skipping (Gromak et al., 2003).

Raver1, which is expressed in most tissue types, can be found not only in the nucleus but also the cytoplasm, where it interacts with cytoskeletal proteins (Hüttelmaier et al., 2001). The protein has three N-terminal RRMs—although only RRM1 has demonstrable, albeit weak, RNA-binding activity (Lee et al., 2009)—and an extended Pro-rich C terminus that contains four conserved PTB-Raver1 interacting motifs (PRIs) with the consensus sequence [S/G][I/L]LGxxP (Rideau et al., 2006; Figure 1A). These motifs, which are essential for Raver1 function, bind exclusively to the α-helical side of the PTB RRM2 opposite the RNA-binding surface, a mode of interaction that permits formation of ternary PTB-RNA-Raver1 complexes. The initial analysis of PTB-Raver1 interactions showed that only PRI1 and PRI3 bind with relatively high affinity (Rideau et al., 2006). Raver2, which is a related protein of unknown function, has a similar domain structure to Raver1: three N-terminal RRMs and a Pro-rich C terminus (Figure 1A; Kleinhenz et al., 2005). Although the C terminus is the least well-conserved portion between the two proteins, Raver2 contains two PRI motifs that are very similar to the PRI1 and PRI3 motifs found in Raver1 and have been shown also to mediate binding to PTB (Henneberg et al., 2010).

The first structural analysis of the interaction of peptides containing Raver1 PRI sequences with PTB only yielded a nuclear magnetic resonance (NMR)-restrained docking model because the affinity of purified PTB RRM2 for synthetic PRI3 peptides was too low for a full structure determination (Rideau et al., 2006). Although it provides valuable insights, this model is not precise enough to allow full dissection of the structural basis of binding of Raver1 PRIs to PTB. By fusing Raver1 PRIs as N-terminal extensions to PTB RRM2, we have now obtained crystal structures of PTB RRM2 complexed with Raver1 PRI3 and PRI4, which are high-affinity and low-affinity motifs, respectively. In combination with mutagenesis, binding, and splicing assays, these new structural data reveal a mode of PTB-Raver1 interaction that is applicable to PTB paralogues and other PRI-containing proteins and that places useful constraints on models of the joint action of PTB and Raver proteins.

## Results

### Construct Design and Characterization

To determine the structure of a PTB-Raver1 complex, we overcame the weak binding of short Raver1 peptides to PTB (Rideau et al., 2006) by fusing the PRI3 sequence as an N-terminal extension of PTB RRM2 to increase the local concentration artificially, a strategy that has worked for other protein-peptide complexes (Candel et al., 2007). The PTB-Raver1 docking model indicated that a linker of at least 20 amino acids would be required to join the C-terminal end of the bound PRI3 peptide to the N terminus of RRM2 (Rideau et al., 2006). The first chimeric construct (PRI3-RRM2) was therefore designed to contain the 12-residue PRI3 sequence (PGVSLLG*A*PPKD—the conserved core residues, which we number 1-7, are underlined) followed by residues 156-285 of PTB RRM2. Residues 156-179 are from the polypeptide that links RRM1 to RRM2 in the full-length protein; residues 180-285 correspond to the structured RRM2 domain (Simpson et al., 2004; see Experimental Procedures). The PRI3 sequence used in the chimera contains a Glu to Ala mutation at position 5 in the core sequence, a carryover from the previous NMR analysis, but this substitution does not affect binding (Rideau et al., 2006).

The expression levels in *E. coli* and the solubility of the PRI3-RRM2 chimera are much higher than for constructs just containing RRM2 from PTB. PRI3-RRM2 is soluble to at least 25 mg/ml, whereas recombinant RRM2 precipitates above 6 mg/ml (Simpson et al., 2004). Although NMR analyses and size-exclusion chromatography suggested that PRI3-RRM2 exhibited concentration-dependent oligomerization (data not shown) the fusion protein produced diffraction-quality crystals. We therefore used the same strategy to fuse PRI1, PRI2, and PRI4 of Raver1 and the PRI from hnRNP-L and matrin-3 to PTB RRM2 (Figure 3D). All these constructs had enhanced solubility similar to PRI3-RRM2, but only the construct containing the low-affinity PRI4 (SSEGLLGLGPGP) also crystallized.

### Crystal Structures of PRI3-RRM2 and PRI4-RRM2

PRI3-RRM2 and PRI4-RRM2 crystals diffracted X-rays to 1.4 Å and 1.55 Å, respectively. Diffraction data were phased by molecular replacement using the NMR structure of PTB RRM2 (Simpson et al., 2004) as a search model. In each case, only central portions of the PRI sequences were revealed by difference electron density maps (PRI3: VSLLG*A*PP; PRI4: SEGLLGL) (see Figure S1 available online); there was no density for the linker peptides connecting them to the RRM2 domain, so these were not incorporated into the atomic models. Final models for PRI3-RRM2 and PRI4-RRM2 were refined to R_free_ values of 23.1% and 22.1%, respectively (see Table 1 for full statistics).

The crystals of PRI3-RRM2 and PRI4-RRM2 have two complexes in the asymmetric unit that have almost identical structures (e.g., for PRI3-RRM2, the all atom root-mean-square deviation [rmsd] is 0.22 Å; Figure S1B). Comparison of the RRM2 structures in each complex with the solution structures of PTB RRM2 (rmsd = 1.0 Å over C_α_ atoms; Simpson et al., 2004; Oberstrass et al., 2005) reveals no significant structural changes upon peptide binding.

### Conformations of PRI3 and PRI4 Bound to PTB RRM2

The crystal structures of PRI3 and PRI4 from Raver1 in complex with PTB RRM2 are consistent with many of the features found in previous work: the peptides bind to the dorsal helical face of the RRM domain and in the same orientation as determined by NMR methods (Figure 1B) (Rideau et al., 2006). However, they provide much more detailed information on the peptide-RRM interaction and reveal previously undetected features of the bound peptide. Strikingly, although our NMR-restrained docking model assumed an extended conformation of the bound peptide, the core peptides of PRI3 and PRI4 in the crystal structures adopt S-shaped conformations that wrap around the peptide-binding surface on RRM2, which is formed by the α1 and α2 helices as well as the β1-α1 and α2-β4 loops (Figures 1C and 1D). Our earlier NMR docking model for the Raver1-RRM2 complex was derived from eleven intermolecular NOEs involving methyl groups and aromatic rings (Rideau et al., 2006). All NOEs to Raver1 methyl groups are satisfied by the crystal structure presented here, with the exception of the A503, which is slightly farther away from the RRM domain than in solution (Figure S2). This small difference is not unexpected, as extensive conformational exchange was observed in NMR spectra of bound Raver1 and likely reflects some averaging in this region in solution.

**Figure 2:**
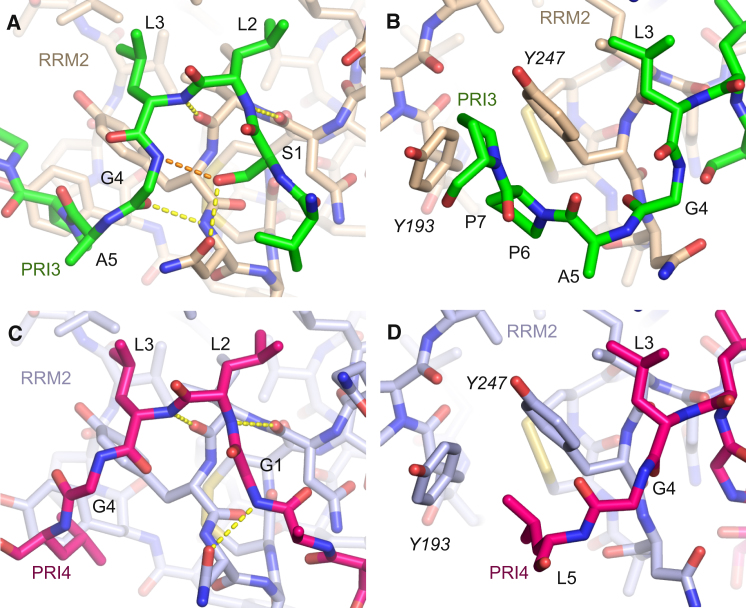
Detailed Structural Comparison of Raver1 PRI3 and PRI4 Bound to PTB RRM2 (A and B) Close-up views of PRI3 bound to RRM2. Intramolecular hydrogen bonds are shown as orange dashes; intermolecular hydrogen bonds are yellow. (C and D) Equivalent views of PRI4 bound to RRM2.

There is an extensive bipartite hydrophobic interface between the peptides and RRM2: the pair of Leu side chains at positions 2 and 3 in the motif both project into a shallow apolar depression between the two helices on the dorsal face of RRM2, whereas downstream residues of the PRIs are packed around the side chains of Tyr 247 and Tyr 193, though in very different conformations for the two peptides (Figure 2). For PRI3, the four-residue sequence ^3^LGAP^6^ wraps around Tyr 247 (Figure 2B), whereas in PRI4 a different backbone conformation means that just three residues, ^3^LGL^5^, are in contact with the tyrosine (Figure 2D). Moreover, although in PRI3 Pro 6 and Pro 7 both contact the side chains of Tyr 247 and Tyr 193, the equivalent residues in PRI4 (Gly 6 and Pro 7) are not visible in the electron density map, presumably due to disorder; this difference is likely to contribute to the lower affinity of PRI4. The occlusion of hydrophobic features on the surface of RRM2 by the Raver1 peptide probably accounts for the enhanced solubility of the chimeric PRI-RRM2 proteins.

The observed hydrophobic contacts made by the Leu side chains at positions 2 and 3 in the PRI explain why substitution of either residue by smaller apolar residues is detrimental to binding (Rideau et al., 2006). However, it is not clear why a Leu-to-Ile substitution at position 2 in PRI3 has no effect, whereas the same substitution at position 3 effectively abrogates binding of the Raver1 PRI, especially since Leu 3 binds within a shallower depression. Perhaps the branching at the C_β_ in Ile introduces a steric clash that distorts nearby hydrogen bonds between the Raver1 PRI3 and PTB RRM2.

In addition to the apolar contacts, there are specific hydrogen bond interactions that contribute to PRI binding to PTB RRM2. These show clear differences between PRI3 and PRI4, which probably also contribute to the affinity differences between these two motifs. In PRI3 (core sequence: SLLGAPP), Leu 2 and Leu 3 both project in the same direction into the binding pocket on PTB RRM2 because of a pinched backbone conformation that is stabilized by an internal hydrogen bond from the side-chain hydroxyl of Ser 1 to the backbone amide of Gly 4 (Figure 2A). In this conformation, the peptide is able to make four hydrogen bonds to PTB RRM2.

In contrast, PRI4 (core sequence: GLLGLGP) makes only three intermolecular hydrogen bonds. The absence of Ser at position 1 eliminates the intrapeptide hydrogen bond and results in a more open backbone conformation. The loss of this internal interaction allows the peptide bond between Leu 3 and Gly 4 to flip with respect to PRI3, a conformational change that eliminates a hydrogen bond to RRM2 (from the carbonyl group of Gly 4; Figure 2C), which likely reduces the affinity of this motif, although the peptide flip is also needed to allow the ^3^LGL^5^ sequence to wrap around Tyr 247.

### Dissecting the Structural Basis of Differential PRI Affinity

Previous work established that PRI1 and PRI3 bind with significantly higher affinity than PRI2 and PRI4 (Rideau et al., 2006). The new structural information suggests that the Leu 2-Leu 3 dipeptide found in both motifs is not sufficient for high-affinity binding; instead, variations in amino acids at positions 1, 5, and 6 appear to be crucial. To explore this idea, we introduced mutations into PRI3 and PRI4 peptides (fused to MS2 proteins at their C termini) and tested their effect on binding affinity in pull-down assays (see Experimental Procedures).

**Figure 3:**
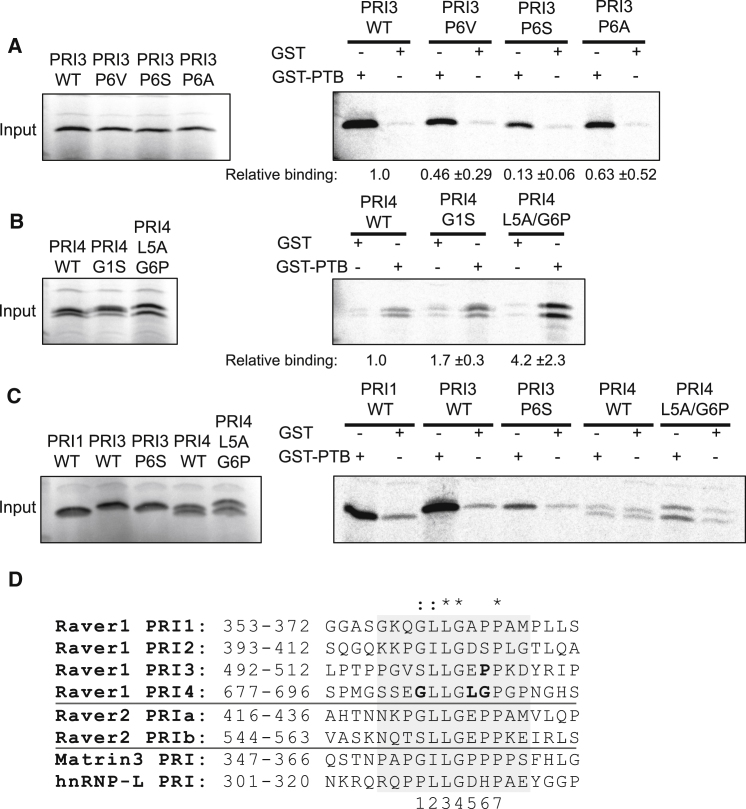
Effect of Mutations of Raver1 PRIs on Binding to GST-PTB (A) Pull-down assays of binding of Raver1 PRI3 mutants to GST-PTB. Left: Loading controls for ^35^S-Met-labeled PRI3-MS2 fusion proteins containing PRI3 wild-type (WT) and the mutants P6V, P6S, and P6A (see Experimental Procedures). Right: Autoradiogram of PRI3-MS2 proteins pulled down with GST (1 μg) or GST-PTB (3 μg). (B) Pull-down assays of binding of Raver1 PRI4 mutants to GST-PTB. Left: Loading controls for ^35^S-Met-labeled PRI4-MS2 fusion proteins. Right: GST pull-down of PRI4 constructs with GST (5 μg) or GST-PTB (15 μg). Although PRI4 constructs migrate as a doublet (as observed previously [Rideau et al., 2006]), both products of the in vitro transcription-translation reaction contain the PRI since they bind PTB with the same affinity. (C) Comparison of binding of wild-type and mutant Raver1 PRIs to GST-PTB. Left: Loading controls. Right: GST pull-down of PRI4 constructs with GST (2 μg) or GST-PTB (6 μg). (D) PRI sequences from murine Raver1 (AAP33691), murine Raver2 (NP_898845), human matrin-3 (NP_001181884), and human hnRNP-L (NM_001533). Sequences shown for Raver1 are the 20 amino peptides used in pull-down assays; the shaded box indicates the sequences included in PRI-RRM2 chimeras for structural studies. Sequence similarity and identity within the PRI core are indicated. Residues in Raver1 PRI3 and PRI4 that were tested by mutagenesis are in boldface. See also Figure S3.

Pro 6 of PRI3, which is conserved in the other high affinity motif, PRI1, makes apolar contacts with Tyr 247 and Tyr 193 that are likely to contribute significantly to binding affinity. This was confirmed by mutagenesis: although conservative mutations of Pro 6 to Ala or Val—both of which retain the apolar character of the side chain—only very slightly reduced the affinity of PRI3 for PTB RRM2, substitution by a polar Ser reduced binding 7-fold (Figure 3A). Thus, the presence of a polar residue at position 6 within the PRI is detrimental to binding, a result that is consistent with the low affinity of Raver1 PRI2 and the PRI from hnRNP L, which have Ser and His at this position, respectively (Rideau et al., 2006) (Figure 3D).

To further explore the peptide features that affect binding to PTB, we performed experiments to examine what changes would be necessary to enhance the binding affinity of Raver1 PRI4. The structure shows that the presence of Gly 1 in PRI4 eliminates the internal stabilization of the backbone of PRI3 due to the hydrogen bond between Ser 1 and Gly 4 (Figures 2A and 2C). However, this interaction seems to have little effect on the affinity for RRM2, because substitution of Gly 1 by Ser in PRI4 only increased the binding 1.7-fold (Figure 3B), consistent with the observation that Gly occurs at position 1 in the high-affinity PRI1 sequence (Figure 3D). In contrast, there was a stronger 4-fold enhancement of binding when the double mutation Leu5Ala/Gly6Pro was used to convert the ^3^LGLGP^7^ sequence in PRI4 to the ^3^LGAPP^7^ found in our modified version of the high-affinity PRI3 and in PRI1 (Figure 3B). Together these observations suggest that interaction of Pro 6 with the tyrosine pocket in PTB RRM2 is essential for a high-affinity interaction with PTB, whereas the backbone stabilization due to Ser 1 plays a minor supporting role. Moreover, the mutagenesis results help to verify the functional relevance of the structures obtained from our artificial chimeric constructs.

Intriguingly, the L5A/G6P substitutions in PRI4 make its core sequence identical to that of PRI1 (Figure 3D), but this mutant binds approximately 10-fold less well to GST-PTB than PRI1 (Figure 3C). This suggests that elements outside the conserved core motif of Raver1 may play a role in binding to PTB. However, although the 20-residue peptide sequence used in the binding assays is longer than the 12-residue sequence incorporated into the chimeric constructs used for crystallization, comparison of the flanking sequences (Figure 3D) reveals no obvious patterns of conservation that correlate with binding.

### Interactions of Raver1 PRIs with nPTB

To identify whether the mode of binding of Raver1 PRI peptides to PTB is the same in other PTB paralogues, we investigated their binding to GST-nPTB in pull-down assays. The sequence identity between PTB and nPTB is over 74% (Markovtsov et al., 2000; Polydorides et al., 2000). Mapping of the RRM2 sequence differences between PTB and nPTB onto the structure reveals that they cluster in two distinct regions. One group is located on the upper surface of helix α2, quite separate from the PRI binding site, whereas a second smaller cluster occurs on the β1-α1 loop, which, together with the adjacent α2-β4 loop, forms part of the Raver1 binding surface (Figure S3A). Despite these differences, binding assays revealed that the relative affinities of PRIs 1-4 for PTB and nPTB are very similar. Thus, PRI1 and PRI3 bind strongly to both paralogues, whereas PRI2 and PRI4 have much weaker affinity (Figure S3C). Furthermore, mutations designed to reduce or enhance the affinities of PRI3 and PRI4 for PTB RRM2 had similar effects on their binding to nPTB: the P6S mutation in PRI3 P6S reduced binding for nPTB, whereas the binding PRI4 L5A/G6P was enhanced (compare Figure S3C with Figure 3). These observations support the contention that PTB and nPTB interact with Raver1 PRIs in the same way.

### PTB Mutations Affect PRI Binding and Activity

To extend our understanding of the PTB-Raver1 interaction, we generated PTB RRM2 mutants and tested them for binding of Raver1 and RNA and for their ability to regulate splicing of *Tpm1*.

**Figure 4:**
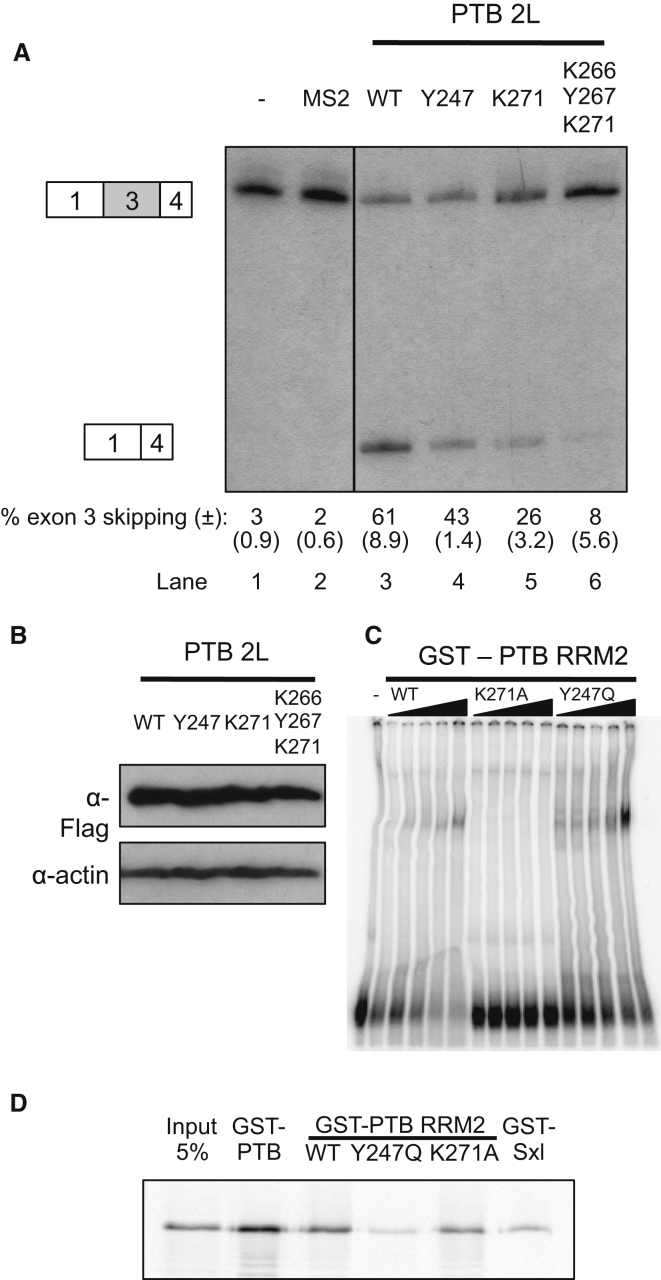
Mutation of PTB RRM2 RNA and PRI Interacting Surfaces Impairs Activity (A) Effects of RRM2 mutations on an MS2-tethered splicing regulation assay. The *Tpm1* exon 1-3-4 splicing reporter, with the PTB site downstream of exon 3 replaced by a pair of MS2 coat protein binding sites, was transfected into HeLa cells, and splicing patterns were analyzed by RT-PCR. Lane 1: reporter alone. Lane 2: reporter cotransfected with MS2 coat protein. Lanes 3-6: reporter cotransfected with MS2-PTB2L expression constructs (wild-type [WT], Y247Q, K271A, K266A/Y267A/K271A, respectively). Percent exon skipping (±SD) is shown below each lane. (B) Western blots to show equivalence of expression of MS2-PTB2L tested in panel A (anti-FLAG). Loading control, anti-actin. (C) Electrophoretic mobility shift assay of 0.2-3.2 μM recombinant PTB RRM2 wild-type (lanes 2-6), K271A (lanes 7-11), and Y247Q (lanes 12-16). Lane 1: no protein. (D) Pull-down of in vitro translated full-length Raver1 with the indicated GST-fusion proteins.

PTB RRM2, along with the following inter-RRM linker (PTB 2L), was previously shown to be fully active as a splicing repressor domain when fused to MS2 coat protein and tethered to *Tpm1* RNA by an MS2 binding site, which replaced the natural downstream PTB binding site (Robinson and Smith, 2006). We therefore used this tethered repressor domain assay to test the effects of mutations designed to target the PTB-Raver1 interaction. Cotransfection of the splicing reporter construct with an MS2 expression vector had no effect on the low basal level of exon skipping (Figure 4A, lanes 1,2), while transfection of wild-type PTB 2L-MS2 led to 61% exon skipping (lane 3). Mutations of Tyr 193, Leu 241, and Gln 244 had no effect (data not shown). However, mutation of Tyr 247 to Gln reduced activity by a third (Figures 4A, lane 4). This reduction in activity is consistent with the close contacts made by Tyr 247 with hydrophobic side chains in each of the PRIs (Figures 2B and 2D). As a control, we also tested the effects of mutations on the RNA-binding surface of RRM2 (K271A and K266A/Y267A/K271A), which are predicted to have no effect on PRI binding. Both single and triple RNA binding mutations severely impaired the splicing repressor activity (Figures 4A and 4B). To confirm the specificity of the mutations, recombinant GST-PTB RRM2 proteins were tested for the ability to pull down in vitro-translated Raver1 protein (Figure 4D) and to bind to RNA (Figure 4C). As expected, the Y247Q mutant showed complete impairment of Raver1 interaction (Figure 4D; compare to GST-Sxl nonspecific control), but bound to RNA comparably to wild-type RRM2. In contrast, the K271A mutant was impaired for RNA binding but interacted with Raver1. These observations are consistent with the previous finding that PTB RRM2 can form a ternary complex with the Raver1 PRI and a short RNA oligomer (Rideau et al., 2006), and confirm the independence of the two interacting surfaces of RRM2. Moreover, it is interesting to note that, given that RRM1 and RRM2 of PTB have been shown to interact with U1 stem-loop IV (Sharma et al., 2011), the strong effect of the RNA-binding mutations in PTB 2L-MS2 on repression of *Tpm1* exon 3 (Figure 4) may be due to impairment of binding to U1 snRNA.

## Discussion

In this report, we present, to our knowledge, the first structural analysis of a PTB-protein complex involved in splicing: the crystal structures of PTB RRM2 in complex with two peptide motifs from Raver1, the protein recruited to corepress exon 3 of *Tpm1*. Though the structures contain only a single domain of PTB, this is the major structured portion of PTB-2L, the minimal fragment required to recapitulate the activity of the full-length protein in exon exclusion (Robinson and Smith, 2006) and, as has been shown more recently, inclusion (Xue et al., 2009; Llorian et al., 2010).

Analysis of the structure and RNA-binding properties of PTB have helped to inform ideas about how PTB works, first by delineating the unexpected architecture of the domains and so paving the way for more precise functional studies using structure-based mutagenesis (Conte et al., 2000; Simpson et al., 2004; Oberstrass et al., 2005), then by revealing modes of RNA binding, which led to testable suggestions of how binding of PTB might remodel RNA to affect splicing (Oberstrass et al., 2005; Petoukhov et al., 2006; Lamichhane et al., 2010).

Our growing understanding of PTB-RNA interactions has influenced models of PTB-mediated exon repression, its best-characterized activity. Several plausible mechanisms have emerged, built on the observation that PTB molecules are involved at multiple binding sites in pre-mRNA located within or near to regulated exons. Possible modes of repression include masking of splicing signals by direct contact (Singh et al., 1995; Wagner and Garcia-Blanco, 2001) or PTB-mediated looping (Chou et al., 2000; Oberstrass et al., 2005; Cherny et al., 2010; Lamichhane et al., 2010; Llorian et al., 2010); additionally or alternatively, PTB may interfere with spliceosome assembly by binding to stem-loop IV of U1 snRNA (Sharma et al., 2011).

Less attention has been paid to the interactions that PTB makes with other proteins to regulate splicing (or indeed, any other PTB-mediated activity), despite observations of PTB-binding partners that have accumulated over the past 10 years and now include not only Raver1 and Raver2 (Hüttelmaier et al., 2001; Henneberg et al., 2010), but also Nova-1 and Nova-2 (Polydorides et al., 2000) and, possibly, MRG15 (Luco et al., 2010). Clearly delineation of the details of PTB-protein interactions is important for a more complete understanding of the molecular mechanism of splicing regulation.

**Figure 5:**
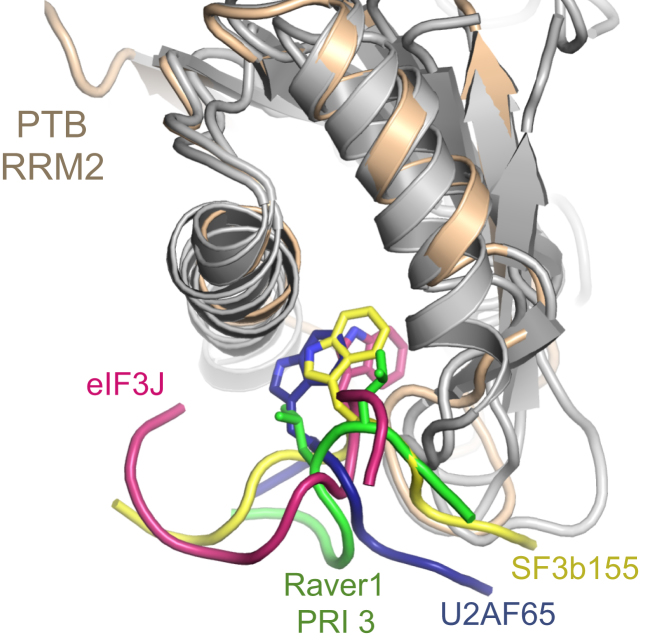
Comparison of the PTB-Raver1 Interaction with Other Peptide-RRM Complexes A common mode of binding is revealed by superposition of the complex of Raver1 PRI3 and PTB RRM2 with the peptide/RRM complexes of U2AF65/U2AF35 (PDB-1jmt), SF3b155/SPF45 (PDB-2peh) and eIF3j/eIF3b (PDB-2krb). PTB RRM2 is colored tan; other RRMs are colored gray. The superposition was performed for the RRM domains using PyMOL (Delano, 2002). See also Figure S4.

The crystal structures of PTB RRM2 in complex with Raver1 reveals a mode of peptide binding that has been observed in other RRM-peptide complexes such as U2AF35/U2AF65 (Kielkopf et al., 2001), SPF45/SF3b155 (Corsini et al., 2007), and eIF3b/eIF3j (Elantak et al., 2010): the peptide binds in an orientation that is broadly perpendicular to the two helices on the dorsal surface of the RRM (Figure 5). The REF/ICP27 complex, in which the peptide lies parallel to the helices, is a notable exception (Tunnicliffe et al., 2011; Figure S4).

Nevertheless, the PTB-Raver1 complex marks an interesting variation on this theme. Although in the three examples of perpendicular binding mentioned above peptide binding is anchored by insertion of a Trp side chain from the peptide into a deep apolar pocket between the helices on the RRM, in the Raver1 peptide the role of this Trp is taken by a pair of Leu side chains that insert into a shallower hydrophobic depression in a similar location on the RRM. This pair of Leu side chains make important hydrophobic contacts that contribute to binding of Raver1 PRIs to PTB RRM2; however, they are not sufficient for high-affinity binding, because this sequence feature is also found in PRI2 and PRI4, which have lower affinity (Rideau et al., 2006). Additional interactions are also important for tight binding. The PRI3-RRM2 complex reveals that these interactions include a number of specific hydrogen bonds—mostly between main-chain groups—and the interaction of the Pro-Pro dipeptide at positions 6 and 7 in the motif with the Tyr pocket formed by Tyr 247 and Tyr 193 (Figure 2B). Although PRI4 exhibits an alternative mode of binding, which inserts a hydrophobic Leu side chain (from position 5 of the motif) within this Tyr pocket, this is insufficient for high-affinity binding (Figure 3C). Moreover, mutagenesis experiments show that substitution of the Pro at position 6 in the PRI3 motif with a polar residue (such as Ser) is sufficient to impair binding (Figure 3A), a result that accounts for the low affinity observed for the PRI2 sequence of Raver1 (Rideau et al., 2006). The overall mix of interactions observed echoes similar observations from other structures of RRM-peptide complexes such as U2AF35/U2AF65 (Kielkopf et al., 2001) and SPF45/SF3b155 (Corsini et al., 2007).

Given the close sequence similarity between the PRI1 and PRI3 motifs from Raver1, the structural and binding data presented here offer a plausible explanation for the high affinity of PRI1 and suggest strongly that it binds to PTB RRM2 in the same way. The same can probably also be said of the two high-affinity PRI motifs in Raver2, which are similar to Raver1 PRI1 and PRI3 (Henneberg et al., 2010; Figure 3D).

We have also shown that despite a cluster of sequence differences between PTB and its neuronal paralogue near the Raver1-binding site on RRM2, nPTB exhibits a very similar pattern of variation of affinity for the Raver1 PRIs (Figure S3). The Raver1 binding site is therefore common to both these paralogues, and it will be worth investigating whether this function is retained by other PTB paralogues such as ROD1 (Yamamoto et al., 1999; Hüttelmaier et al., 2001) and smPTB (Gooding et al., 2003).

Furthermore, the results allow us to refine the definition of what constitutes a high-affinity PRI sequence from [S/G][I/L]LGxxP to [S/G][I/L]LGxΦP, where the Φ at position 6 indicates a preference for a small hydrophobic residue (Pro, Val, Ala). Using ScanProsite (de Castro et al., 2006), 36 human proteins can be identified that contain predicted high-affinity PRIs conforming to [S/G][I/L]LGx[AVP]P, including Raver1 and Raver2, each of which contain two sites. The nuclear matrix protein matrin-3 contains the motif GILGPPP, which is necessary and sufficient for interaction with PTB (M.C. and C.W.J.S., unpublished data). In addition, the 3′ end processing factors CSTF2 and CSTF2T both contain the motif GLLGDAP suggesting a molecular basis for how PTB is able to activate some polyadenylation sites (Castelo-Branco et al., 2004). Other potential PRI containing proteins such as the deacetylase HDAC6 and a histone demethylase, JMJD8, hint at further interesting and functionally diverse targets of PTB.

The PTB-Raver1 complexes presented here place constraints on possible modes of corepression by PTB and Raver1. Although there are four PTB-binding motifs in the C-terminal region of Raver1, each appears capable of only binding to a single molecule of PTB. The stoichiometry of PTB-Raver1 complexes that assemble on regulated exons could therefore be 2:1, if only high-affinity sites are engaged. However, it remains possible that low-affinity sites may also contribute to binding, not least because tethering of Raver1 to PTB via the two high-affinity sites will augment the local concentration of PRIs. This would be consistent with the model envisaged by Cherny et al. (2010) on the basis of their observations of multiple PTB molecules binding in the vicinity of regulated exons in *Tpm1*. Against this, however, it is has been observed that mutation of a single PRI3 motif in Raver1—and of an identical PRI in Raver2 (PRIb in Figure 3D)—was sufficient to abrogate binding to PTB and PTB-mediated localization to perinucleolar comparments in HeLa cells (Henneberg et al., 2010). Although this may point to a 1:1 stoichiometry for PTB-Raver1 complexes, it remains true that mutation of other high-affinity PRIs in Raver1 or Raver2 significantly reduces binding to PTB (Rideau et al., 2006; Henneberg et al., 2010). The full details of functional PTB-Raver1 interactions have yet to be worked out. It is interesting to note the similar spacing between the high-affinity PRIs in Raver1 and Raver2 (approximately 135 amino acids; see Figure 1A), which perhaps points to a common architecture of functional PTB-Raver complexes.

## Experimental Procedures

### Plasmid Construction

For structural analysis, PRI-RRM2 chimera constructs containing residues 156-285 of PTB were generated by PCR using PTB1 complementary DNA as a template (Gil et al., 1991). The forward primer incorporated an NcoI site and sequences coding for 12 amino acids from the PRI sequences (from Raver1, hnRNP-L, and matrin-3), whereas the reverse primer introduced a stop codon and downstream HindIII site. The resulting PCR product was ligated into the pETM-11 vector, which adds a TEV^pro^-cleavable N-terminal 6xHis tag (Zou et al., 2003).

For pull-down experiments, we used Quikchange (Stratagene) to mutate MS2-Raver1 fusion proteins that were made previously (Rideau et al., 2006). These incorporate 20-residue Raver1 PRI sequences as N-terminal extensions. Plasmids for GST-PTB (Gromak et al., 2003) and GST-nPTB—a gift from B.J. Blencowe—(Calarco et al., 2009) have been described. All plasmids were sequenced by MWG Eurofins Ltd.

### Protein Expression and Purification

PTB proteins were expressed at 37°C in *E. coli* BL21 (DE3). Cell pellets were lysed by sonication in Buffer A (250 mM NaCl, 25 mM Tris [pH 7.8]), 0.1% (v/v) Triton X-100, and 0.5 mM phenylmethanesulfonylfluoride containing 1 mg/ml lysozyme. 6xHis-tagged proteins were purified from clarified lysates using TALON resin (Clontech). Purified proteins were eluted in Buffer A containing 100 mM imidazole. The 6xHis tag was removed by overnight incubation at 4°C with 1 mg of his-tagged TEV^pro^ per 30 mg of PRI-RRM2 during dialysis against Buffer A with 5 mM β-mercaptoethanol. The cleaved tag and TEV^pro^ were removed in a second round of TALON purification. For crystallization, proteins were further purified by gel filtration on a Superdex 75 16/60 column (GE Healthcare) in 100 mM NaCl, 25 mM Tris (pH 7.8), and 1 mM dithiothreitol (DTT).

For pull-down assays to monitor Raver1 binding, glutathione S-transferase (GST) tagged PTB proteins were extracted from *E. coli* in a similar manner and applied a glutathione sepharose 4B column (GE Healthcare). The column was washed with Buffer A and proteins were eluted in Buffer A + 5 mM DTT and 20 mM glutathione (pH 9.0). Purified proteins were concentrated by centrifugal filtration to 22 mg/ml, 28 mg/ml, 5.5 mg/ml, and 4.3 mg/ml for PRI3-RRM2, PRI4-RRM2, GST-PTB, and GST-nPTB, respectively and stored at −80°C.

GST-PTB RRM2 fusion proteins used in Figure 4 were PCR amplified and cloned into the EcoRI site in pGEX3 (to incorporate residues 181-284). Cells were lysed by passing twice through a French press in MTPBS buffer (150 mM NaCl, 16 mM NaH_2_PO_4_, 1 mM DTT, 3 mM PMSF, EDTA-free protease cocktail [Roche]). Soluble and insoluble fractions were separated by centrifugation at 8,000 g for 10 min. GST fusion proteins were purified through a glutathione sepharose 4B column (GE Healthcare), washed with MTPBS + 1% Triton X-100, and step-eluted using reduced glutathione. Protein-containing fractions were dialyzed in Buffer E (20 mM HEPES, 100 mM KCl, 3 mM MgCl_2_, 0.1 mM EDTA, 1 mM DTT, 20% glycerol, 0.05% Nonidet-P40). The GST-PTB and GST-SXL proteins used as a control in Figure 4D were prepared as described (Rideau et al., 2006).

### Structure Determination

Purified recombinant PRI-RRM2 proteins at ∼18 mg/ml were crystallized by sitting drop vapor diffusion using a reservoir solution containing 0.2 M NaI, 0.1 M Bis-Trispropane (pH 6.5), and 20% polyethylene glycol 3350. Crystals were soaked for 1 min in mother liquor with 20% glycerol before flash freezing in liquid N_2_. X-ray diffraction data were collected at beamlines X13 at DESY, Hamburg, Germany, and I02 at Diamond Light Source, Didcot, UK. The data were processed with iMosfilm and scaled using SCALA from the CCP4 suite (Collaborative Computational Project, No. 4, 1994). PRI3-RRM2 data were phased by molecular replacement with Phaser v1.2 (McCoy et al., 2007) using the ensemble of NMR structures of PTB RRM2 as a search model (Simpson et al., 2004). PRI4-RRM2 was phased by rigid body refinement using the RRM2 domain from the refined PRI3-RRM2 structure since the two chimeric proteins crystallized isomorphously. Models were manually adjusted in O (Jones et al., 1991) and refined with CNS (Brünger et al., 1998).

### Pull-Down Assays

In vitro GST pull-down assays were performed essentially as described in (Gromak et al., 2003). Briefly, ^35^S-Met-labeled MS2-Raver1 proteins produced by in vitro transcription-translation reactions (TNT Quick Coupled System, Promega) were incubated for 3 hr at 4°C with GST, GST-PTB, or GST-nPTB in wash buffer (100 mM KCl, 20 mM HEPES [pH 7.9], 0.5 mM DTT, 0.2% Tween-20, and 10% glycerol) in the presence of 5 μl glutathione sepharose 4B. Beads were then washed three times with wash buffer, and bound protein was eluted using SDS loading buffer for SDS-PAGE analysis. Protein band intensities on dried gels were recorded using a Fuji FLA-5000 phosphor imager; quantitative densitometry was performed using AIDA software (Raytest).

### Electrophoretic Mobility Shift Assays

GST-PTB RRM2 proteins (0.2-3.2 μM) were incubated with 10 fmol of an RNA probe spanning the *Tpm1* exon 3 polypyrimidine tract, which is enriched in PTB binding sites (Cherny et al., 2010) in 10 mM HEPES, 100 mM KCl, 3 mM MgCl_2_, 5% glycerol, 1 mM DTT, 0.25 μg of *E. coli* rRNA, RNase inhibitor (DCP) for 30 min at 30°C and loaded directly onto a 5% polyacrylamide (40:1) gel.

### Splicing Assays

Splicing transfection assays were carried out as described previously (Robinson and Smith, 2006). Briefly, 200 ng of the splicing reporter and 800 ng of effector DNA were transfected into HeLa cells in 35-mm wells, followed by RT-PCR analysis of the splicing products 48 hr after transfection (Robinson and Smith, 2006). The splicing reporter pT2Δbp-2MS2 is a modified version of TM-2MS2 (Robinson and Smith, 2006) containing a mutation of the canonical branch-point of the *Tpm1* exon 3, which leads to enhanced exon skipping in HeLa cells (Gooding et al., 2006).

## Figures and Tables

**Table 1:** Data Collection, Data Processing, and Refinement Statistics for Crystal Structures of PRI3-RRM2 and PRI4-RRM2

	PRI3-RRM2	PRI4-RRM2
Diffraction data		
Space group	C2	C2
a, b, c (Å)	74.23, 60.60, 60.84	74.48, 60.38, 61.06
α, β, γ (°)	90, 90, 107.51	90, 90, 107.86
Resolution range (Å)	30.32-1.40 (1.48-1.40)	35.49-1.55 (1.63-1.55)
Reflections	50129	36156
Multiplicity	5.6 (5.3)	2.4 (2.4)
Completeness (%)	98.9 (96.1)	96.9 (96.5)
I/σ_I_	15.4 (5.3)	10.2 (3.0)
R_merge_ (%)	6.4 (35.4)	5.5 (35.8)
Refinement statistics		
R_calc_ (%)	22.3	21.6
R_free_ (%)	23.1	22.1
Nonhydrogen atoms	1741	1723
Waters	191	132
Rms bond lengths (Å)	0.005	0.005
Rms bond angles (°)	1.32	1.31
Ramachandran (% favored/allowed)	98.0/1.4	97.7/2.3
PDB ID	3zzy	3zzz

Values in parentheses are for the highest resolution shell. PDB, Protein Data Bank; Rms, root-mean-square.

## Supplemental Information

Document S1. Four Figures
