# Supplementary material for: Crystallographic Analysis of Polypyrimidine Tract-Binding Protein-Raver1 Interactions Involved in Regulation of Alternative Splicing
Source: Structure. 2011 Dec 7;19(12):1816–25. doi: 10.1016/j.str.2011.09.020 (PMC3420021; doi:10.1016/j.str.2011.09.020)
Supplement: Document S1. Four Figures [file mmc1.pdf]

Structure, Volume *19*

Supplemental Information

Crystallographic Analysis of Polypyrimidine Tract-Binding Protein-Raver1 Interactions Involved in Regulation of Alternative Splicing

Amar Joshi, Miguel B. Coelho, Olga Kotik-Kogan, Peter J. Simpson, Stephen J. Matthews, Christopher W.J. Smith, and Stephen Curry

Inventory of Supplemental Information

Figure S1. Crystallographic results for Raver1 PRIs bound to PTB RRM2.
This relates to Figure 1 and provides additional detail on the structure by (i) showing the electron density for the bound peptides in the two structures and (ii) providing a superposition of the bound peptide conformations for the two complexes in the asymmetric unit of the crystal in each case. The figures therefore provide the reader with data to gauge their confidence in the structure.

Figure S2. Comparison of the X-ray structure with NOEs measured in solution.
This supplementary figure does not relate specifically to a particular figure in the manuscript but is relevant to the description of the structure at the beginning of the Results section in which we discuss the new crystal structures (presented in our paper) with our previously published NMR-restrained docking model. This figure was included at the suggestion of one of the reviewers to provide a demonstration that the earlier docking model is consistent with the crystal structure, which it is.

Figure S3. PTB and PTB homologues have the same affinity for PRIs.
This figure relates to the discussion in the text of the binding of Raver1 peptides to closely-related paralogues of PTB. In panel A are shown (on the structure of PTB) the locations of amino acid substitutions (also visible in the sequence alignment in panel B) on the peptide binding face of RRM2 in these paralogues. This illustrates (as discussed in the text) the conservation of the part of the surface that binds the Raver1 peptide. In support of these insights, experimental measurements of the interaction of Raver1 peptides with nPTB are shown in panels C and D. These data are important for substantiating the claim that Raver1 binds to nPTB and, in all likelihood, to other PTB paralogues.

Figure S4. Superposition of the structure of the PRI3-RRM2 complex with the ICP27/REF peptide-RRM complex.
This figure relates to Figure 5 in the manuscript. It shows an unusual peptide-RRM complex, in which the orientation of the bound peptide is perpendicular to the mode that is more commonly observed. We could not include this structure in Figure 5 because it would have become too cluttered but thought it worthwhile to include as supplementary information.

Figure S1 (related to Fig. 1): Crystallographic results for Raver1 PRIs bound to PTB RRM2.

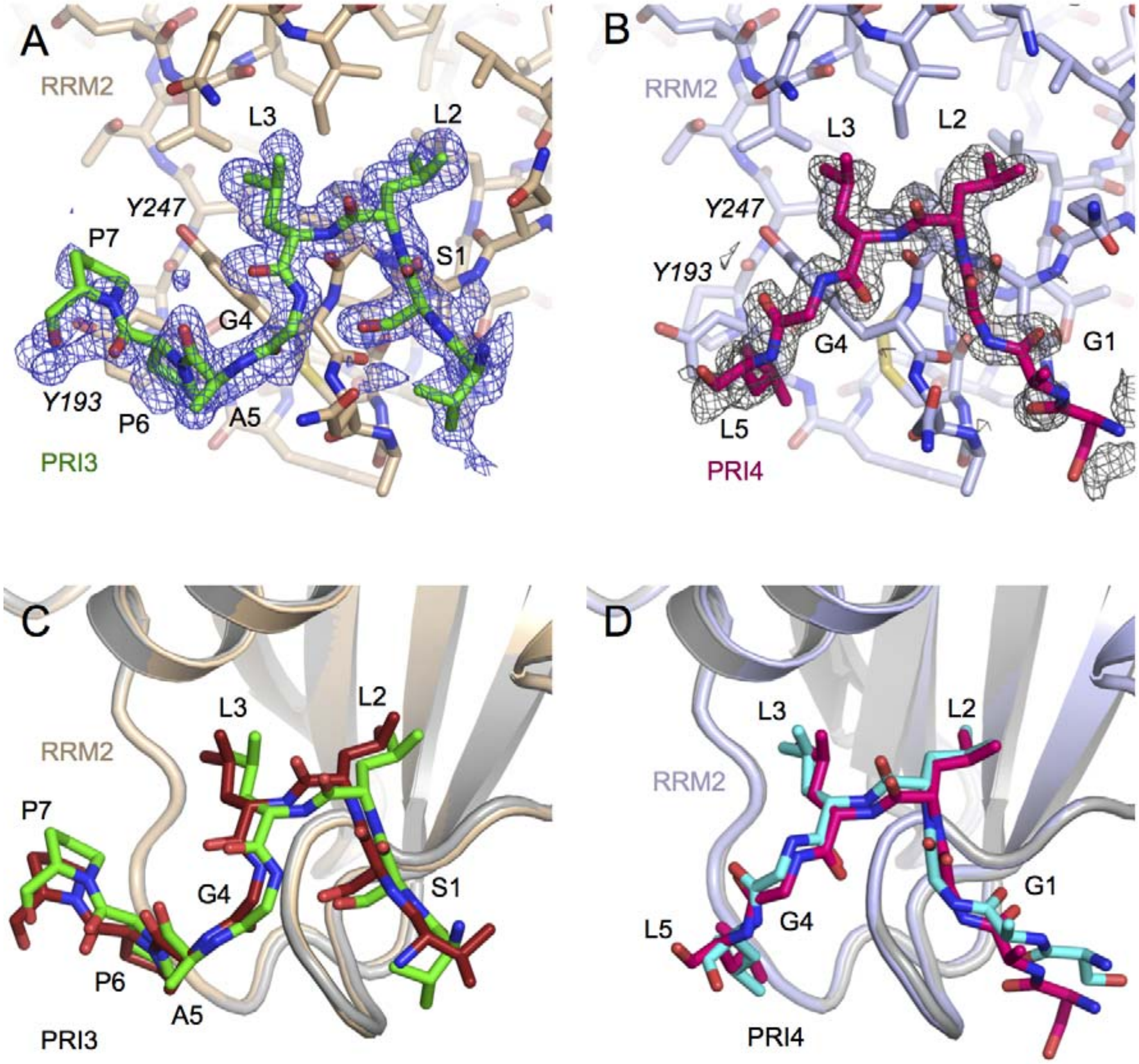

Simulated annealing F_o-F_c omit maps of (A) PRI3-RRM2 and (B) PRI4-RRM2, both phased in the absence of a model for the PRI peptide. (C) Comparison of molecules A and B from the crystal asymmetric unit of PRI3. (D) Comparison of molecules A and B from the crystal asymmetric unit of PRIs 3 and 4 respectively.

Figure S2: Comparison of the X-ray structure with NOEs measured in solution.

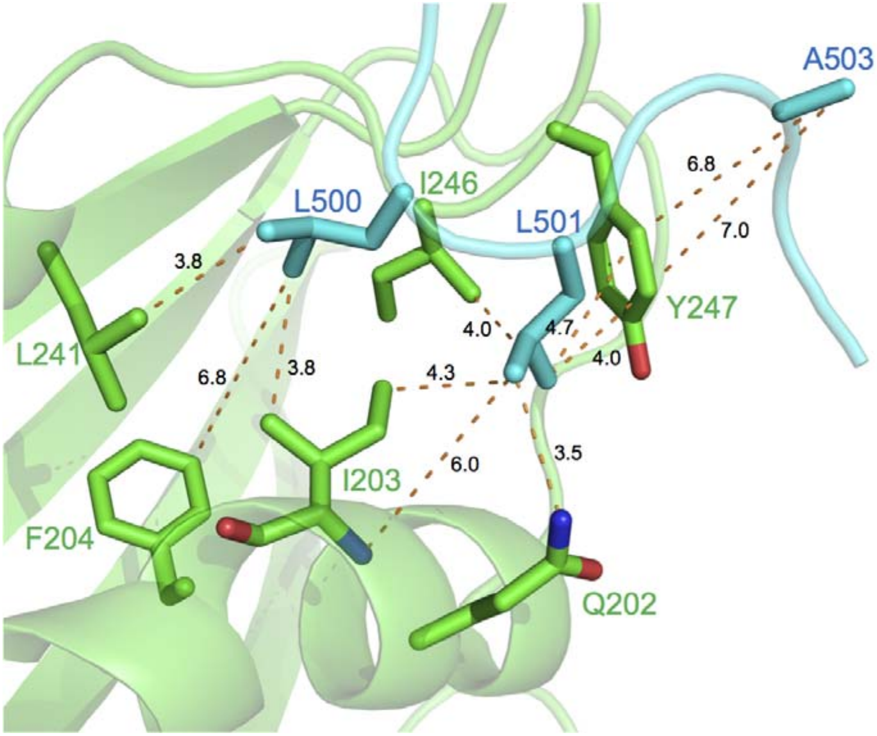

The 11 NOEs observed in the previous NMR study of the RRM2 complex with Raver1 peptide (Rideau et al., 2006) are shown as dashed lines on the X-ray structure determined here. RRM2 is coloured green, Raver1 cyan, with key side-chains drawn as sticks. The distances shown are carbon-carbon or carbon-nitrogen and are taken from the crystal structure. As the NOEs are between hydrogen atoms, in most cases the distances shown greatly overestimate the actual distance measured by NMR. The structure is thus consistent with the NMR data, with the exception of the position of Ala 503 on Raver1, which on average is closer in the dynamic solution ensemble detected by NMR that was observed in the crystal structure. This discrepancy may be due to increased mobility of the bound peptide in the solution structure, as compared to the crystallised complex.

Figure S3 (Related to Fig. 3): PTB and PTB homologues have the same affinity for PRIs.

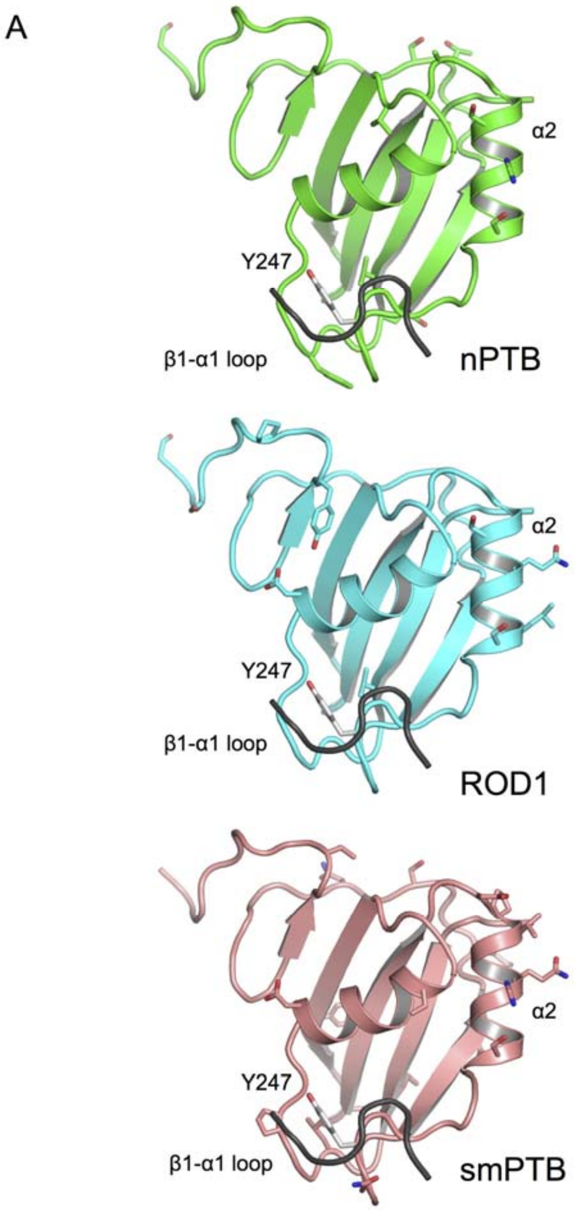

(A) Mapping of sequence differences between PTB and nPTB, ROD1 and smPTB onto the structure of the Raver1-PTB complex (PRI3-RRM2). Residues from PTB that are altered in the three paralogues are shown as sticks. Y247, which is conserved between the paralogues, is also shown.

Figure S3 (Related to Fig. 3; cont.): PTB and PTB homologues have the same affinity for PRIs. (cont.)

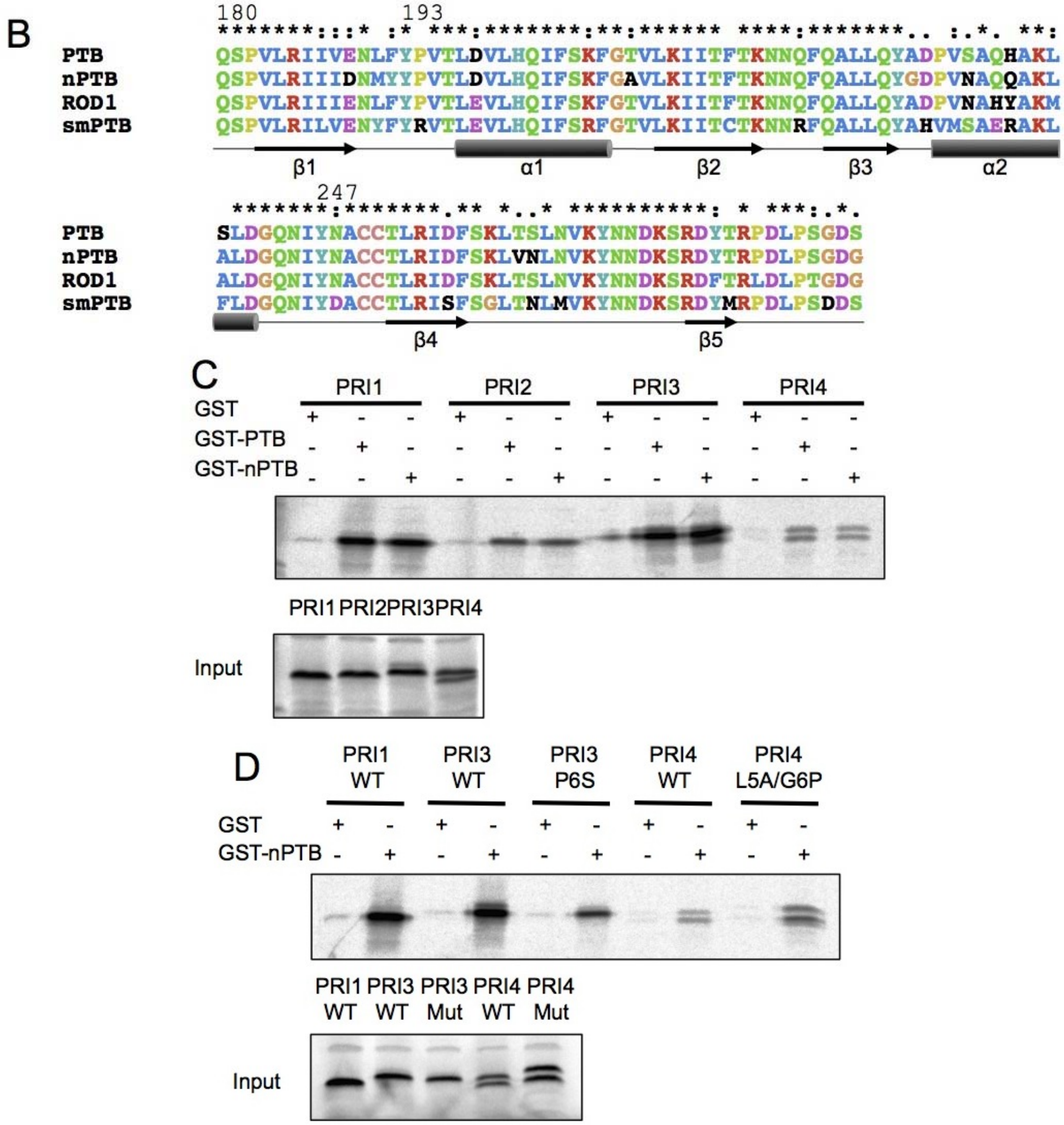

(B) Alignment of RRM2 sequences for PTB paralogues. (C) GST-pulldown of Raver1 PRIs with 2 µg GST or 6 µg GST-PTB or GST-nPTB. (D) GST pulldown of Raver1 PRIs 1, 3 and 4 and mutants (PRI3 P6S and PRI4 L5A/G6P) with 2 µg GST or 6 µg GST-nPTB.

Figure S4 (Related to Fig. 5): Superposition of the structure of the PRI3-RRM2 complex with the ICP27/REF peptide-RRM complex.

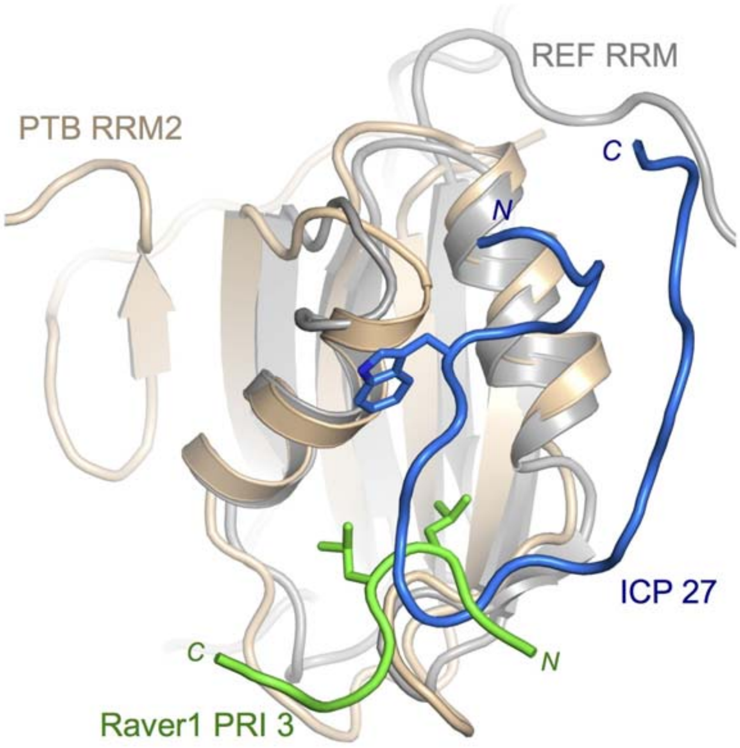

The Raver1 peptides (green) bind perpendicular to the helices of PTB RRM2 (tan) inserting two leucines (shown as sticks) into a shallow hydrophobic pocket. In contrast, the peptide from ICP27 (blue) binds in an orientation that is *parallel* to dorsal helices in the REF2 RRM (grey) (Tunnicliffe et al., 2011). The ICP27/REF complex nevertheless displays some similarity with other peptide/RRM complexes since the ICP27 peptide inserts a tryptophan side chain into a hydrophobic pocket between the helices to stabilise the interaction.

References

Rideau, A.P., Gooding, C., Simpson, P.J., Monie, T.P., Lorenz, M., Hüttelmaier, S., Singer, R.H., Matthews, S., Curry, S., and Smith, C.W.J. (2006). A peptide motif in Raver1 mediates splicing repression by interaction with the PTB RRM2 domain. Nat. Struct. Mol. Biol. *13*, 839-848.

Tunnicliffe, R.B., Hautbergue, G.M., Kalra, P., Jackson, B.R., Whitehouse, A., Wilson, S.A., and Golovanov, A.P. (2011). Structural basis for the recognition of cellular mRNA export factor REF by herpes viral proteins HSV-1 ICP27 and HVS ORF57. PLoS Path. *7*, e1001244.
